# *Anaplasma phagocytophilum* Manipulates Host Cell Apoptosis by Different Mechanisms to Establish Infection

**DOI:** 10.3390/vetsci3030015

**Published:** 2016-07-15

**Authors:** Pilar Alberdi, Pedro J. Espinosa, Alejandro Cabezas-Cruz, José de la Fuente

**Affiliations:** 1 SaBio. Instituto de Investigación en Recursos Cinegéticos IREC (CSIC-UCLM-JCCM), Ciudad Real 13005, Spain; espinosaprados.pedroj@gmail.com; 2 Center for Infection and Immunity of Lille (CIIL), INSERM U1019-CNRS UMR 8204, Université Lille Nord de France, Institut Pasteur de Lille, Lille 59000, France; cabezasalejandrocruz@gmail.com; 3 Department of Veterinary Pathobiology, Center for Veterinary Health Sciences, Oklahoma State University, Stillwater, OK 74078, USA

**Keywords:** tick, *Anaplasma*, apoptosis, neutrophil, immunology, tick-borne diseases, *Ixodes*

## Abstract

*Anaplasma phagocytophilum* is an emerging zoonotic pathogen that causes human and animal granulocytic anaplasmosis and tick-borne fever of ruminants. This obligate intracellular bacterium evolved to use common strategies to establish infection in both vertebrate hosts and tick vectors. Herein, we discuss the different strategies used by the pathogen to modulate cell apoptosis and establish infection in host cells. In vertebrate neutrophils and human promyelocytic cells HL-60, both pro-apoptotic and anti-apoptotic factors have been reported. Tissue-specific differences in tick response to infection and differential regulation of apoptosis pathways have been observed in adult female midguts and salivary glands in response to infection with *A. phagocytophilum.* In tick midguts, pathogen inhibits apoptosis through the Janus kinase/signal transducers and activators of transcription (JAK/STAT) pathway, while in salivary glands, the intrinsic apoptosis pathways is inhibited but tick cells respond with the activation of the extrinsic apoptosis pathway. In *Ixodes scapularis* ISE6 cells, bacterial infection down-regulates mitochondrial porin and manipulates protein processing in the endoplasmic reticulum and cell glucose metabolism to inhibit apoptosis and facilitate infection, whereas in IRE/CTVM20 tick cells, inhibition of apoptosis appears to be regulated by lower caspase levels. These results suggest that *A. phagocytophilum* uses different mechanisms to inhibit apoptosis for infection of both vertebrate and invertebrate hosts.

## 1. Introduction

*Anaplasma phagocytophilum* is an obligate intracellular bacterium transmitted primarily by *Ixodes* spp. ticks with a great impact on both human health and animal production worldwide [1,2]. *A. phagocytophilum* is a cocoid, gram negative bacterium that infects host immune cells, mainly neutrophils, and endothelial cells of vertebrate hosts. Inside the cell, it forms dense intracellular microcolonies called morulae [3]. *A. phagocytophilum* has a wide host range that coincides with the widespread distribution of the tick vectors [4,5,6]. Different genetic variants of *A. phagocytophilum* have been reported which vary in host preferences, host responses, and tick vectors, with differences between ruminant and human/dog strains [4,7,8,9]. The disease was first described in small ruminants in Europe—particularly sheep—as the aetiologic agent of tick-borne fever (TBF) [10]. Diseases caused by *A. phagocytophilum* include human granulocytic anaplasmosis (HGA) and equine and canine granulocytic anaplasmosis [1,2]. Tick larvae acquire the pathogen from small wild rodents; it is transstadially transmitted to nymphs and adults that infect a new mammalian host during a subsequent bloodmeal [1,3]. The pathogen is not transovarially transmitted and needs to cycle between the mammalian host and the arthropod vector to survive [3].

Intracellular bacteria infecting host immune cells have evolved to avoid destruction within the cells. The mechanisms employed by these intracellular pathogens include diverse molecular pathways to invade cells and manipulate host defence mechanisms [11,12]. Apoptotic cell death is a highly complex innate immune mechanism designed to maintain cell populations in tissues. It is also activated in response to microbial infection that results in reduction of infected cells, thus benefiting the remaining cells [13]. However, intracellular bacteria use different strategies to inhibit cell apoptosis in order to enhance their replication and survival [13,14]. Like other intracellular bacterium, *A. phagocytophilum* has evolved mechanisms to subvert host response to facilitate infection, multiplication, and transmission [15]. These molecular mechanisms include remodelling of the cytoskeleton, manipulation of the immune response, control of host cell epigenetics and delay of cell apoptosis to complete the developmental cycle in vertebrate neutrophils and tick cells [15,16,17,18,19,20,21].

*A. phagocytophilum* employs common strategies to infect vertebrate host and tick cells, including the inhibition of cell apoptosis, but the infection also affects mechanisms involving other genes and proteins that have been described only in vertebrate host cells [15]. Signalling processes leading to apoptosis are classified into two main pathways—the extrinsic or death receptor mediated apoptosis pathway and the intrinsic or mitochondria mediated apoptosis pathway [22]—both of which lead to the activation of a caspase cascade that converge at the level of mitochondria [23]. There is an additional pathway known as the perforin/granzyme pathway that involves T-cell mediated cytotoxicity and perforin-granzyme-dependent killing of the cell that has also been identified in *I. scapularis* ticks except for the presence of the perforin ortholog [24]. Additionally, different biological processes could lead to apoptotic cell death [1,15,24,25,26,27].

In this review, we focused on the effect of *A. phagocytophilum* infection on the inhibition of cell apoptosis, which appears to be a key adaptation mechanism to facilitate infection and survival of *A. phagocytophilum* in both vertebrate hosts and ticks.

## 2. *A. phagocytophilum* Inhibits Apoptosis in Human Cells

**Figure 1:**
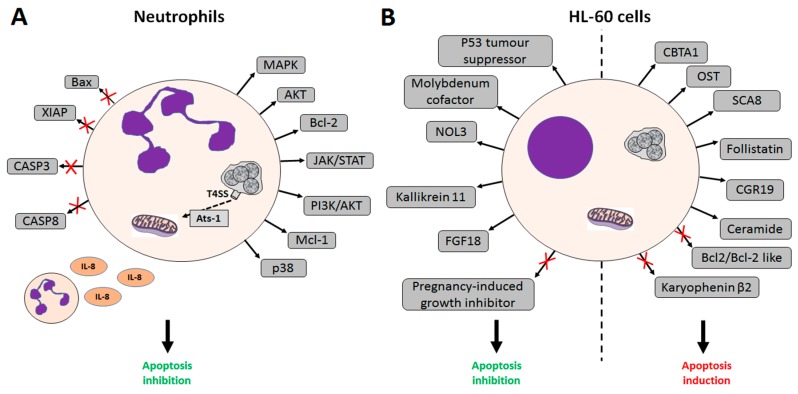
Effects of *A. phagocytophilum* infection on apoptosis pathways in human cells. (**A**) Effects of *A. phagocytophilum* infection on apoptosis in human neutrophils. *A. phagocytophilum* secretes the bacterial Type IV secretion system (T4SS) substrate Ats-1 that reaches the mitochondria, inhibiting cytochrome c release, inhibits Bax translocation into the mitochondria, and induces the expression of anti-apoptotic factors. Granulocyte macrophage colony-stimulating factor (GM-CSF)/cytokine stimulation of neutrophils results in activation of the Janus kinase/signal transducers and activators of transcription (JAK/STAT) pathway, resulting in the increase of Mcl-1protein levels and bacterial viability. The *bcl-2* gene is activated, X-linked inhibitor of apoptosis protein (XIAP) degradation induced, and caspase-3 activation blocked. phosphoinositide kinase-3/protein kinase B (PI3K/AKT) kinases and IL-8 secretion are activated, stimulating the recruitment of neutrophils; (**B**) Effects of *A. phagocytophilum* infection on apoptosis in HL-60 cells. Factors with anti-apoptotic effect up-regulated in infected cells include genes coding for P53 tumour suppressor mutant gene, molybdenum cofactor sulphurase, nucleolar protein 3 (NOL3), kallikrein 11, and fibroblast growth factor 18 (FGF18). The pregnancy-induced growth inhibitor is down-regulated in infected cells. The pro-apoptotic effect in infected cells is supported by up-regulation of the calmodulin-binding transcription activator 1 (CBTA-1), the heparan sulphate (glucosamine) OST, the human spinocerebellar ataxia type 8 (SCA8), follistatin, the cell growth regulator 19 (CGR19), and ceramide. The karyopherin (importin) beta 2 and the anti-apoptotic factors Bcl-2 and Bcl-2-like proteins are down-regulated in infected cells.

*A. phagocytophilum* is emerging as a human pathogen in the U.S., Europe and Asia [28], where HGA is a potentially life-threatening disease [2]. Infection of *A. phagocytophilum* in humans has been detected in neutrophils and bone marrow progenitor cells, and to a lesser extent in monocytes and macrophages [16]. Neutrophils are short-lived and develop a strong oxidative stress response towards pathogens. Inhibition of neutrophil apoptosis is an essential mechanism in HGA and appears to be triggered by surface and/or secreted *A. phagocytophilum* proteins [26,27,29,30]. *A. phagocytophilum* infection delays neutrophil apoptosis in vivo [29,31] and infected neutrophils maintain high mitochondrial membrane potential compared to uninfected cells [32]. *A. phagocytophilum* has the ability to inhibit both extrinsic and intrinsic apoptotic pathways in neutrophils at multiple levels. *A. phagocytophilum* inhibits the intrinsic pathway of the spontaneous neutrophil apoptosis by protecting the mitochondrial membrane integrity [32]. Bacteria delay apoptosis by secreting Ats-1, an effector molecule secreted by *A. phagocytophilum* Type IV secretion system (T4SS) [26,27]. Ats-1 translocates inside the mitochondria to inhibit apoptosis through inhibition of both cytochrome c release and poly ADP-ribose polymerase (PARP) cleavage [26,27]. The bacterium inhibits Bax translocation (a pro-apoptotic member of the Bcl-2 family) into the mitochondria and induces the expression of anti-apoptotic genes [33]. Activation of the *bcl-2* gene and degradation of the X-linked inhibitor of apoptosis protein (XIAP, a member of the IAP family of proteins and a direct inhibitor of caspases), alongside with inhibition of mitochondria-mediated apoptotic caspase-3 processing and cleavage of pro-caspase-8, caspase-8, and caspase-9 have been reported [32,33]. *A. phagocytophilum* also activates the Janus kinase/signal transducers and activators of transcription (JAK/STAT) pathway and kinases such as the phosphoinositide kinase-3 (PI3K)/protein kinase B (Akt) and p38 MAP kinase signalling [34,35]. *A. phagocytophilum* regulates the expression of the anti-apoptotic protein myeloid leukemia cell differentiation Mcl-1, thus inhibiting apoptosis and stimulating IL-8 autocrine secretion, leading to the recruitment of neutrophils [1,32,33]. *A. phagocytophilum* infection up-regulates the expression of anti-apoptotic members of the Bcl-2 family, blocks anti-CD95-induced programmed cell death in human neutrophils, and blocks clustering of CD95 at the cell surface during spontaneous neutrophil apoptosis, as determined by several microarray analyses [30,33]. Other genes involved in the inhibition of neutrophil apoptosis in vivo include the gene coding for P53 tumour suppressor mutant that is present in cancer cells [36] and is up-regulated in infected cells. These mechanisms allow *A. phagocytophilum* to survive in a host cell-derived vacuole and replicate to develop into morulae within infected human cells [29] (Figure 1).

The rickettsia can be propagated in the laboratory in undifferentiated human promyelocytic cells HL-60 and in HL-60 cells differentiated into neutrophil-like cells, potential precursors of the myelomonocytic lineage [37]. In contrast to neutrophils, infected HL-60 cells appear to be more apoptotic than uninfected cells [16]. Thus, *A. phagocytophilum*-induced apoptosis delay appears to be a neutrophil-specific process and not a global consequence of infection [16]. Nevertheless, microarray analysis of infected HL-60 cells showed changes in genes whose expression had apoptotic and anti-apoptotic effects [38]. For instance, the nucleolar protein 3 (NOL3)—an apoptosis repressor with a caspase recruitment domain—is up-regulated in HL-60 infected cells, whereas anti-apoptotic factors Bcl-2 and Bcl-2-like proteins are down-regulated in infected cells [38], thus suggesting that control mechanisms of cell growth induced by *A. phagocytophilum* infection are quite complex and operate at different levels depending on the host cell type [15] (Figure 1).

## 3. *A. phagocytophilum* Inhibits Apoptosis in Vertebrate Host Cells

*A. phagocytophilum* infects a wide range of hosts, including wild and domestic animals [4,5,6,39], and is the most widespread tick-borne infection in small ruminants in Europe [10]. Genetic analysis has identified the existence of several strains of *A. phagocytophilum*; those isolated from ruminants appear to differ from strains infecting humans and dogs [4,7]. However, sheep can be infected with human strains and are a good model for the study of molecular interactions between ticks and different isolates of *A. phagocytophilum* [40]. Tick feeding site studies have confirmed that sheep experimentally infected with the human NY-18 isolate of *A. phagocytophilum* can be a source of infection for ticks [41]. In human promyelocytic cells, *A. phagocytophilum* infection affects genes involved in essential cellular mechanisms and protective response against infection [38], while in sheep, the infection activates inflammatory and innate immune pathways [42,43].

In mammals, the JAK/STAT pathway is the principal signalling mechanism for a wide array of cytokines and growth factors, which appear to be differentially expressed in infected animals [44]. Activation of the JAK/STAT pathway has also been observed in *A. phagocytophilum*-infected wild boar and sheep when compared to uninfected controls [17]. Up-regulation of host innate immune pro-inflammatory genes and signalling pathways constitutes a general antibacterial mechanism in response to pathogenic intracellular bacteria such as *A. phagocytophilum* [16,42]. *A. phagocytophilum* infection induces both innate and adaptive immunity, indicating that this pathogen circumvents host-cell defences by down-regulating immune genes and delaying the apoptotic death of neutrophils [17]. Adaptive immunity is pathogen-induced through up-regulation of genes such as cluster differentiation 4 (CD4) and IL-21. 

Apoptosis is inhibited in ovine cells as confirmed by an ex vivo study on ovine neutrophils infected in vivo with a sheep isolate of *A. phagocytophilum* [31]. Gene expression profile studies in sheep in response to *A. phagocytophilum* infection showed differences in ruminant hosts [42]. For instance, the apoptosis mediator G-protein linked receptor (*edg-2*) gene was found down-regulated in experimentally-infected sheep, which had not been reported before [42,45]. These differences may be the result of species-specific variations and/or the effect of different pathogen strains [4,42].

In fact, pigs naturally and experimentally infected with *A. phagocytophilum* can control bacterial infection through the activation of innate immune responses and cytoskeleton rearrangement to promote phagocytosis and autophagy [46]. Gene expression profile analysis in naturally-infected pigs did not show an effect on caspases 3 and 8 and the PI3K/AKT pathway, which have been linked to *A. phagocytophilum*-induced apoptosis inhibition in human neutrophils [34,35,46]. Control of *A. phagocytophilum* infection in pigs may result in low infection levels or infection clearance, thus contributing to the low percentage of infection prevalence detected in this species in most regions and suggests that they may have a low impact as reservoir hosts for *A. phagocytophilum* [47]. These results suggest that, although *A. phagocytophilum* evolved mechanisms to subvert innate immune responses and inhibit apoptosis in vertebrate hosts, some species may still activate innate immune protective mechanisms to control infection, therefore highlighting species-specific differences in host response to infection [47].

## 4. *A. phagocytophilum* Inhibits Apoptosis in Ticks and Tick Cells

**Figure 2:**
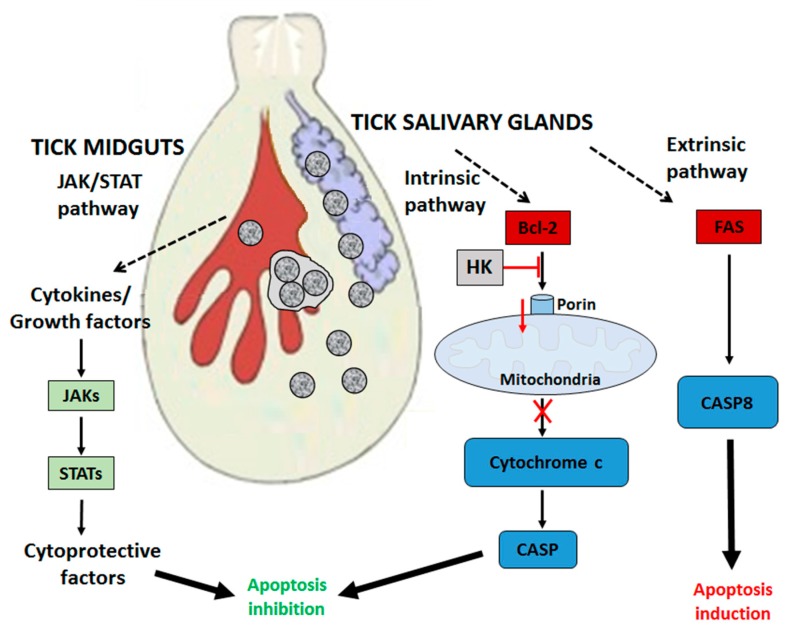
Effect of *A. phagocytophilum* infection on apoptosis pathways in ticks. Tissue-specific differences in response to infection are evident. In tick midguts, *A. phagocytophilum* inhibits apoptosis through up-regulation of the JAK/STAT pathway. In tick salivary glands, down-regulation of porin results in the inhibition of the cytochrome c release, inhibiting the mitochondrially-induced intrinsic apoptosis pathway. This effect is balanced in part by the induction of the extrinsic apoptosis pathway through the inhibition of fatty acid synthase (FAS) proteins. Up-regulated in **green**, down-regulated in **red**. CASP: caspase; HK: hexokinase.

*A*. *phagocytophilum* is transmitted by several tick species [6], ticks of the *Ixodes ricinus* complex, *I. scapularis,* and *I. pacificus* in the USA [2] and *I. ricinus* in Europe [10] being the most important vectors. Tick guts and salivary glands play major but very different roles during pathogen infection, multiplication, and transmission [48,49]. Tick midguts are probably the most important tissue for survival, since they are the initial site of uptake and replication, while the salivary glands are the final place for replication and transmission. The inhibition of cell apoptosis seems to play a central role in *A. phagocytophilum* infection of ticks and tick cells, but tissue-specific differences in tick response to infection and differential regulation of apoptosis pathways have been observed [15,24,50]. Ayllón, et al. [24,51] recently demonstrated that *A. phagocytophilum* infection inhibits cell apoptosis through the activation of the JAK/STAT pathway and inhibits the mitochondrial intrinsic apoptosis pathway to establish infection in *I. scapularis* tick midguts and salivary glands. Reactive oxygen species (ROS)-mediated damage to midgut epithelial cells results in activation of the JAK/STAT pathway, which in turn inhibits apoptosis that facilitates infection of tick salivary glands [24,52]. The JAK/STAT pathway (JAK, STAT, JAK receptor, and suppressor of cytokine signalling proteins SOCS) was down-regulated in response to *A. phagocytophilum* infection of *I. scapularis* nymphs [24], whereas in midgut cells, the JAK/STAT pathway genes encoding JAK, STAT, and JAK-receptor proteins were up-regulated [24]. *A. phagocytophilum* inhibited the intrinsic apoptosis pathway in tick salivary glands by down-regulating porin (voltage-dependent anion-selective channel) expression, resulting in the inhibition of cytochrome c release as the anti-apoptotic mechanism to facilitate bacterial infection [24,51]. Porin is regulated in part by the hexokinase (HK), which also appears down-regulated in infected cells [51]. Thus, bacterial infection induces mitochondrial dysfunction, thereby inhibiting mitochondrial-mediated apoptosis and subverting host cell defences. Ticks respond by activating the extrinsic apoptosis pathway through the inhibition of Fatty Acid synthase (FAS) proteins, thus triggering apoptosis in tick salivary glands to limit bacterial infection and ensure tick survival [24]. Modulation of other molecules such as the X-linked inhibitor of apoptosis (XIAP) E3 ubiquitin ligase [53,54] appears to be required for *A. phagocytophilum* infection in ticks. The α-fodrin (spectrin α-chain) is also involved in *A. phagocytophilum* infection/multiplication and the tick cell response to infection in *I. scapularis* [51]. The pathogen presence decreases expression of α-fodrin in tick salivary glands and porin in both the midgut and salivary glands to inhibit apoptosis, subvert host cell defences, and increase infection. In the midgut, α-fodrin up-regulation was used by the pathogen to increase infection due to cytoskeleton rearrangement that is required for pathogen infection (Figure 2).

**Figure 3:**
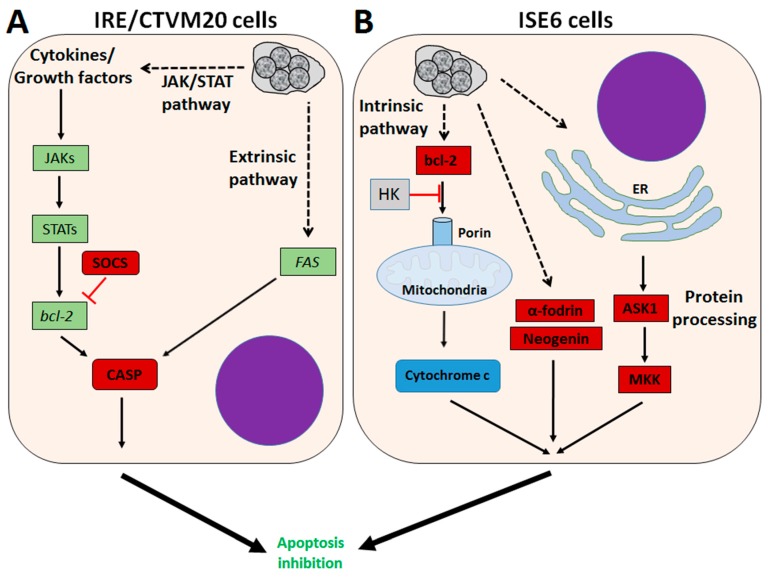
Effect of *A. phagocytophilum* infection on apoptosis pathways in tick cells. (**A**) In *I. ricinus* IRE/CTVM20 cells, *A. phagocytophilum* inhibits apoptosis through up-regulation of the JAK/STAT pathway and up-regulation of FAS coding genes in the extrinsic pathway; (**B**) *A. phagocytophilum* inhibits apoptosis in *I. scapularis* ISE6 cells by down-regulation of the the intrinsic apoptosis pathway, modulation of protein processing in the ER and glucose metabolism. Down-regulation of α-fodrin and Neogenin has also been observed in ISE6 cells. Up-regulated in **green**, down-regulated in **red**. ASK1: apoptosis signal-regulating kinase 1; MKK: mitogen-activated protein kinase; SOCS: suppressor of cytokine signalling.

*A. phagocytophilum* can be maintained in the tick cell lines—IDE8 and ISE6—that were originally derived from *I. scapularis* embryos [55,56,57], and in IRE/CTVM19 and IRE/CTVM20 cell lines, derived from *I. ricinus* embryos [58,59]. Although cultured tick cells have been shown to be a good model for the study of tick-*Anaplasma* interactions, differences exist between cultured tick cells and in comparison with tick tissues in the apoptotic response to pathogen infection [24,50,51,59,60]. The transcriptional response to infection of *I. scapularis* ISE6 cells resembles that of tick hemocytes, while the response in *I. ricinus* IRE/CTVM20 cells appears to be more closely related to that reported previously in infected tick midguts [60]. *A. phagocytophilum* infection inhibits ISE6 tick cell apoptosis through down-regulation of *porin* expression, causing lower cytochrome c protein levels to inhibit the intrinsic apoptosis pathway and facilitate infection [51,59]. Down-regulation of *neogenin* in ISE6 tick cells [60] suggested a new mechanism by which bacterial infection inhibits apoptosis to facilitate infection [61]. However, this mechanism has not been identified in *I. scapularis* nymphs or adult female midguts and salivary glands in response to *A. phagocytophilum* infection [24] (Figure 3).

In *I. ricinus* IRE/CTVM20 tick cells, the inhibition of apoptosis seems to be regulated by lower caspase protein levels in infected tick cells [59]. The transcriptional profile of apoptosis pathway genes obtained from RNAseq data showed down-regulation of *suppressor of cytokine signalling* (SOCS) and up-regulation of *Janus kinase* (JAK) involved in activation of the JAK/STAT pathway, up-regulation of *FAS* implicated in the extrinsic apoptosis pathway, and up-regulation of *cytochrome c* and *bcl-2 interacting protein* of the intrinsic apoptosis pathway [60]. The up-regulation of *FAS* suggests a possible effect of *A. phagocytophilum* infection on the inhibition of the extrinsic apoptosis pathway [60]. Inhibition of the intrinsic apoptosis pathway has been observed in *I. scapularis* tick salivary glands and ISE6 cells, but not in *I. ricinus* IRE/CTVM20 cells [24,51,59,60]. *I. ricinus* IRE/CTVM20 cells appear to be more similar to *I. scapularis* midguts after infection with *A. phagocytophilum* [24,60], which, together with previous results in tick cells [59], suggests a role for the JAK/STAT pathway in the inhibition of apoptosis in *I. ricinus* IRE/CTVM20 infected cells (Figure 3).

In *I. scapularis* ISE6 cells, *A. phagocytophilum* infection affects protein processing in the endoplasmic reticulum (ER) and glucose metabolic pathways by lowering protein levels of phosphoenolpyruvate carboxykinase (PEPCK), mitogen-activated protein kinase (MKK), and apoptosis signal-regulating kinase 1 (ASK1), resulting in the inhibition of tick cell apoptosis in order to increase pathogen infection [50]. *A. phagocytophilum* induces protein misfolding to counteract the tick cell response and facilitate infection, but requires protein degradation to prevent ER stress and cell apoptosis to survive in infected cells [50]. These results suggest tick–pathogen co-evolutionary mechanisms that guarantee the completion of both tick and pathogen life cycles [62]. Additionally, *A. phagocytophilum* may benefit from the tick cell ability to limit rickettsial infection through PEPCK inhibition, leading to decreased glucose metabolism and the availability of essential metabolites for bacterial growth, which also results in the inhibition of cell apoptosis that increases infection of tick cells [50] (Figure 3).

*A. phagocytophilum* infection also manipulates *I. scapularis* tick cell epigenetics, as demonstrated by Cabezas-Cruz, et al. [25]. Their research showed an increase in the levels of histone modifying enzyme (HME) such as p300/CBP, histone deacetylase, and Sirtuin, resulting in inhibition of cell apoptosis that in turn facilitates pathogen infection and multiplication. These results also suggest that a compensatory mechanism might exist by which *A. phagocytophilum* manipulates tick HMEs to regulate transcription and apoptosis in a tissue-specific manner to facilitate infection, but preserving tick fitness to guarantee survival of both pathogens and ticks [62].

## 5. Conclusions

Intracellular bacteria elicit a diverse range of host protective responses. Amongst them, host cell death is critical to influence disease outcome. *A. phagocytophilum* has evolved common molecular mechanisms—including the inhibition of cell death—to establish infection in tick vectors and vertebrate hosts as a result of co-evolution and adaptation to a large number of tick and reservoir host species [5,15,62]. Understanding the manipulation of cell response mechanisms such as apoptosis triggered by *A. phagocytophilum* during host-pathogen and tick-pathogen interactions will provide insights into new strategies for the prevention and control of HGA and TBF.

## Abbreviations

The following abbreviations are used in this manuscript:

HGA: Human Granulocytic Anaplasmosis
TBF: Tick-borne Fever
Ats-1: *Anaplasma* translocated substrate 1 protein
T4SS: Type IV secretion system
PARP: Poly-ADP-ribose polymerase
bcl-2: B cell lymphoma 2
XIAP: X-linked inhibitor of apoptosis protein
JAK/STAT: Janus kinase/signal transducers and activators of transcription
PI3K: phosphoinositide kinase-3
ROS: Reactive oxygen species
AKT: protein kinase B
MAP: mitogen-activated protein kinase
Mcl-1: myeloid leukemia cell
NOL3: nucleolar protein 3
edg-2: G-protein linked receptor
SOCS: suppressor of cytokine signalling proteins
HK: hexokinase
FAS: Fatty Acid Synthase
CASP: caspase
PEPCK: phosphoenolpyruvate carboxykinase
MKK: Mitogen-activated protein kinase kinase
ASK1: apoptosis signal-regulating kinase 1
ER: endoplasmic reticulum
HME: histone modifying enzymes
GM-CSF: Granulocyte macrophage colony-stimulating factor
FGF18: fibroblast growth factor 18
CBTA1: Calmodulin- binding transcription activator 1
OST: Heparan sulphate (glucosamine) OST
SCA8: human spinocerebellar ataxia type 8
CGR19: cell growth regulator 19
